# One-class machine learning classification of skin tissue based on manually scanned optical coherence tomography imaging

**DOI:** 10.1038/s41598-023-28155-5

**Published:** 2023-01-17

**Authors:** Xuan Liu, Samantha Ouellette, Marielle Jamgochian, Yuwei Liu, Babar Rao

**Affiliations:** 1 Department of Electrical and Computer Engineering, New Jersey Institute of Technology, Newark, NJ 07102 USA; 2 Center for Dermatology, Rutgers Robert Wood Johnson Medical School, 1 Worlds Fair Drive, Somerset, NJ 08873 USA; 3 Rao Dermatology, 95 First Avenue, Atlantic Highlands, NJ 07716 USA; 4 Department of Dermatology, Weill Cornell Medicine, 1305 York Ave 9th Floor, New York, NY 10021 USA

**Keywords:** Biomedical engineering, Electrical and electronic engineering

## Abstract

We investigated a method for automatic skin tissue characterization based on optical coherence tomography (OCT) imaging. We developed a manually scanned single fiber OCT instrument to perform in vivo skin imaging and tumor boundary assessment. The goal is to achieve more accurate tissue excision in Mohs micrographic surgery (MMS) and reduce the time required for MMS. The focus of this study was to develop a novel machine learning classification method to automatically identify abnormal skin tissues through one-class classification. We trained a deep convolutional neural network (CNN) with a U-Net architecture for automatic skin segmentation, used the pre-trained U-Net as a feature extractor, and trained one-class support vector machine (SVM) classifiers to detect abnormal tissues. The novelty of this study is the use of a neural network as a feature extractor and the use of a one-class SVM for abnormal tissue detection. Our approach eliminated the need to engineer the features for classification and eliminated the need to train the classifier with data obtained from abnormal tissues. To validate the effectiveness of the one-class classification method, we assessed the performance of our algorithm using computer synthesized data, and experimental data. We also performed a pilot study on a patient with skin cancer.

## Introduction

Nonmelanoma skin cancers (NMSCs), primarily basal cell carcinoma (BCC) and squamous cell carcinoma (SCC), are the most common cancers in the United States^1^. NMSCs can be treated effectively by Mohs micrographic surgery (MMS). In MMS, the tumor is excised and sectioned for immediate histological evaluation in stages to help achieve clear margins with minimal healthy tissue removal. It is used for tumors that are locally invasive and have a high risk of recurrence. During MMS, initial delineation of the tumor is guided by visual inspection and has limited accuracy. Therefore, multiple stages of excision with histological preparation and evaluation are often needed to achieve complete tumor removal, which can keep a patient in the doctor’s office for hours^2^. To achieve more accurate tissue excision (particularly at the first stage) and reduce the time required for MMS, we developed a single fiber optical coherence tomography (OCT) instrument to performed in vivo skin imaging and tumor boundary assessment. The single fiber OCT imaging instrument and artificial intelligent data analysis described here has the potential to impact a wide range of surgical procedures other than MMS, benefiting patients and clinicians.

OCT is a cross-sectional imaging modality that detects scattered light from subsurface tissue microstructures^3,4^. When this scattered light encounters a reference beam, interferometric light is picked up by a detector enabling depth resolved imaging. OCT has a wide range of biomedical applications, most notably in the fields of ophthalmology^5,6^. OCT is advantageous compared to other noninvasive imaging modalities, such as reflectance confocal microscopy (RCM), as it has increased imaging depth (up to several millimeters), and ultrasonography, as it has a higher resolution (≤ 15 μm). Previous studies exploring the application of OCT in dermatology have validated its use for identifying NMSCs^7–11^.However, the utility of conventional OCT for accurately delineating tumor margins prior to surgery is limited by the size of the instrument. Conventional OCT devices rely on mechanical beam scanners to perform lateral scanning. As a result, the probe often covers the entire tumor, making it difficult to mark the tumor boundaries detected through OCT imaging. Therefore, there have been efforts to identify techniques to circumvent this issue, including the use of ink markings or Steri-strips that can be seen on imaging and used as landmarks^12^. However, there currently lacks a mechanism to correlate the tumor edge detected in an OCT image with the precise physical location at the patient’s skin. This led us to develop a single fiber OCT instrument described in our previous study^13^.

We intend to use the single fiber OCT imager to identify the boundaries of biopsy-confirmed tumors, as OCT signals can be used to differentiate between tumor and normal skin. However, extracting clinically relevant information from OCT images to guide MMS is challenging, as pathological features in OCT images are often obscured by speckle noise and depth dependent signal decay/deterioration. Accurate detection of tumor margins on OCT images must be done by an experienced reader, and readers are not usually available in the clinical setting. Moreover, the results of visual inspection depend on the reader’s training in dermatology and pathology, and can vary significantly between individuals. Various methods have been developed to streamline the analysis of skin OCT images, with most methods relying on empirically selected, manually engineered features for tissue classification. In this study, to address the clinical need for accurate skin tissue characterization in MMS, we developed a robust machine learning method that analyzes OCT images and performs automatic skin tissue classification using a unique one-class classification approach. Our method involves extraction of features for tissue classification from a pre-trained deep convolutional neural network (CNN) with a U-Net architecture. The features are used to train the support vector machine (SVM) classifier that performs one-class classification for anomaly detection.

The novelty of this study is the use of neural network as a feature extractor and the use of one-class SVM for abnormal tissue detection. CNNs have found many applications in the processing and analysis of biomedical images. In dermatology, researchers demonstrated deep neural networks that achieved classification accuracy comparable to dermatologists^14^. Compared to manually selected features, CNN features are extracted automatically at different abstract layers, and have the potential to provide a more objective and comprehensive characterization for the tissue in OCT imaging^15^. On the other hand, we adopt a one-class classification strategy to overcome the limited number of images representing abnormal tissue in the training data. Conventionally, the classifier that performs automatic tissue classification (normal versus cancerous or abnormal) undergoes a supervised training process. The classifier is trained using data annotated as normal or abnormal (cancerous). It is essential that the data set used to train the classifier has sufficient examples representing normal and abnormal skin tissues. However, it is challenging to obtain such a comprehensive training data set, because OCT images for skin cancers have significantly different features, depending on the type, stage, and grade of the skin cancer. To overcome the challenge in establishing a data set for supervised training, we train a one-class classifier to recognize normal skin tissue using OCT data obtained from healthy subjects. The one-class classifier has the capability to detect the skin tumor as an anomaly regardless of cancer type, stage, and grade^16,17^.

In summary, this manuscript describes a method that automatically detects skin tumors through OCT imaging. We trained a U-Net for automatic skin segmentation, used the network as a feature extractor, and trained a one-class SVM using these features to detect abnormal tissues. We validated the performance of our algorithm using computed synthesized data, and experimental data. We also performed a pilot imaging experiment on a patient with skin cancer.

## Methods

### OCT imaging system

**Figure 1:**
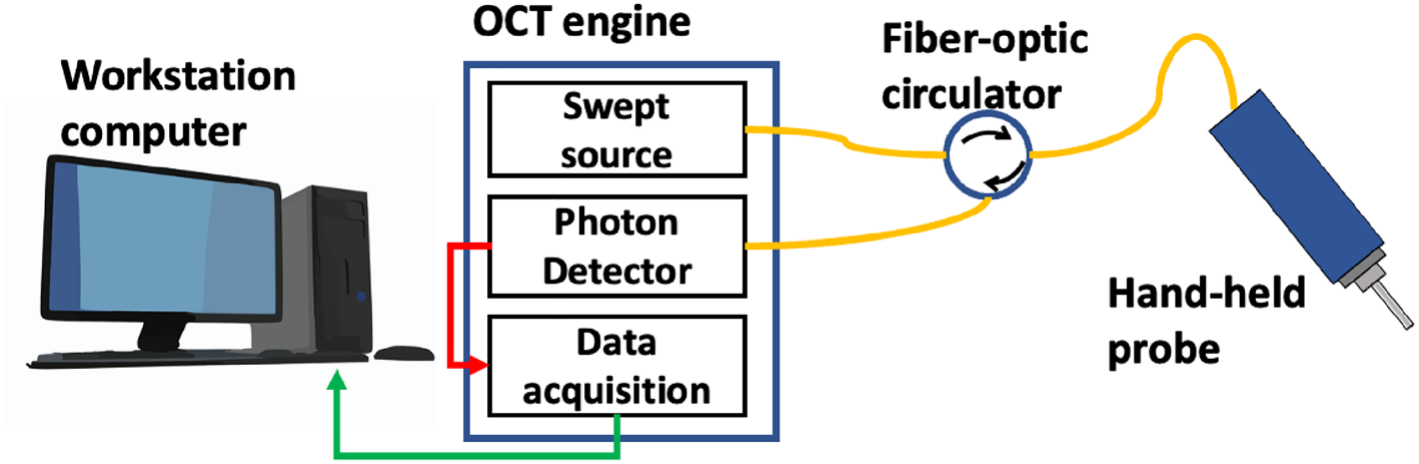
Schematic of manually scanned OCT imaging system.

To perform skin imaging, we utilized an OCT imaging platform described in our previous publications^13^. As illustrated in Fig. 1, the OCT image platform is based on a 1060 nm swept source OCT engine (AXSUN). The output of the swept source is routed by a fiber optic circulator to a single fiber probe. The tip of the cleaved fiber probe provides a reference light (***E***_r_) that interferes with signal light from the sample (***E***_s_). The fiber-optic probe is integrated with a stainless-steel needle with a rubber cap at its tip (20-gauge feed needle, Roboz Surgical Instrument). The metal needle shaft provides mechanical rigidity for the probe. The rubber cap ensures gentle contact between the probe and the skin, and minimizes the deformation of skin layers during scanning. Unlike a conventional OCT imaging system based on a Michelson interferometer, our single fiber probe enables common path OCT imaging where ***E***_r_ and ***E***_s_ share the same probe path. In addition, the manually scanned OCT imaging platform acquires 2D images through manual scanning and performs speckle decorrelation analysis to correct distortion artifacts^18^. The axial sampling interval is approximately 5 μm and the transverse sampling interval is approximately 17 μm.

### U-Net for skin OCT image segmentation

**Figure 2:**
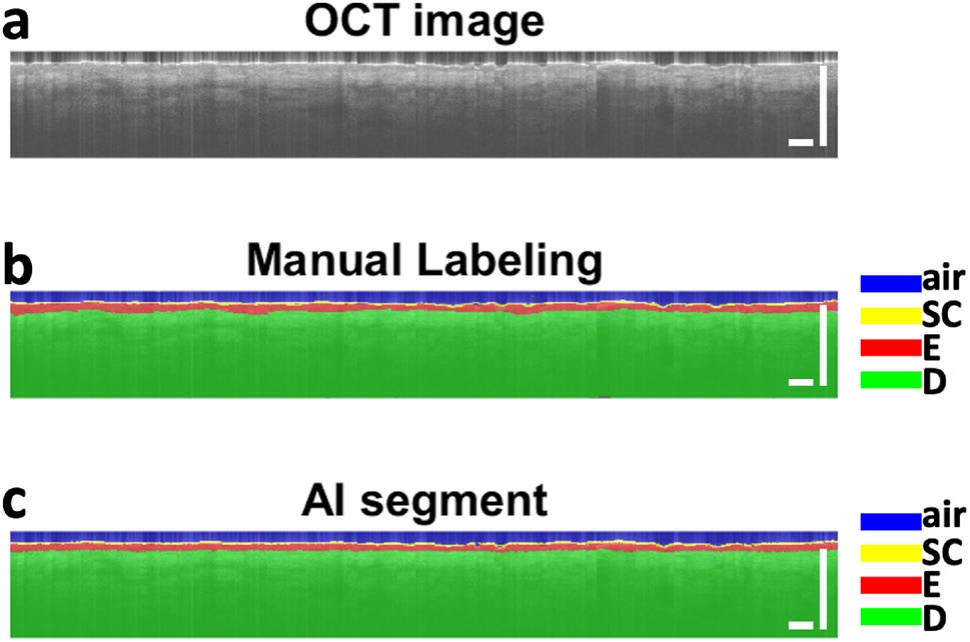
(**a**) OCT image and (**b**) manually labeled layers of air (blue), stratum corneum (SC) (yellow), epidermis (red), and dermis (green) for U-Net training; (**c**) U-net generates labels for individual pixels of the OCT image. Scale bars represent 1 mm.

We trained a U-Net for automatic skin segmentation. The U-Net effectively functionalized the simple single fiber OCT instrument to perform tasks including dermis-epidermis junction (DEJ) assessment and skin layer thickness quantification. In this study, we use the U-Net a feature extractor. U-Net, similar to other deep learning approaches, learns the image features at different scales and different abstraction layers through a training process. The activations of neurons in the layer prior to the segmentation layer are used as the feature for classification in a one-class SVM classifier. The configuration and training strategy of the U-Net is similar to what was described in our previous publications^19,20^. The dimension of input and output images is 256 (axial dimension or z dimension) × 32 (lateral dimension or x dimension). The use of small image patches consisted of 32 Ascans is critical for localized tissue classification. For each pixel, the U-Net effectively calculates its likelihood to be stratum corneum, epidermis, and dermis, and assigns a category accordingly. The network is trained using image data (Fig. 2a) and ground truth pixel classification based on manual annotation (Fig. 2b), and segments an OCT image into difference skin layers (Fig. 2c). The U-Net has a contracting encoder branch and an expanding decoder branch. The encoder branch has five stages to extract multiscale features of the input image while the decoder branch has five stages to generate a spatially resolved pixel category. Each encoder stage has five layers (3 × 3 convolution layer, ReLU activation layer, 3 × 3 convolution layer, ReLU activation layer, and max pooling layer). Each decoder stage consists of seven layers (up convolution layer for upsampling, up ReLU layer, concatenation layer, 3 × 3 convolution layer, ReLU layer, 3 × 3 convolution layer, and ReLU layer). The 1st encoder stage and the last decoder stage generate 16 features that are used for image segmentation and later for SVM classification. Cross entropy is used as the loss function for training.

### SVM for one-class classification

**Figure 3:**
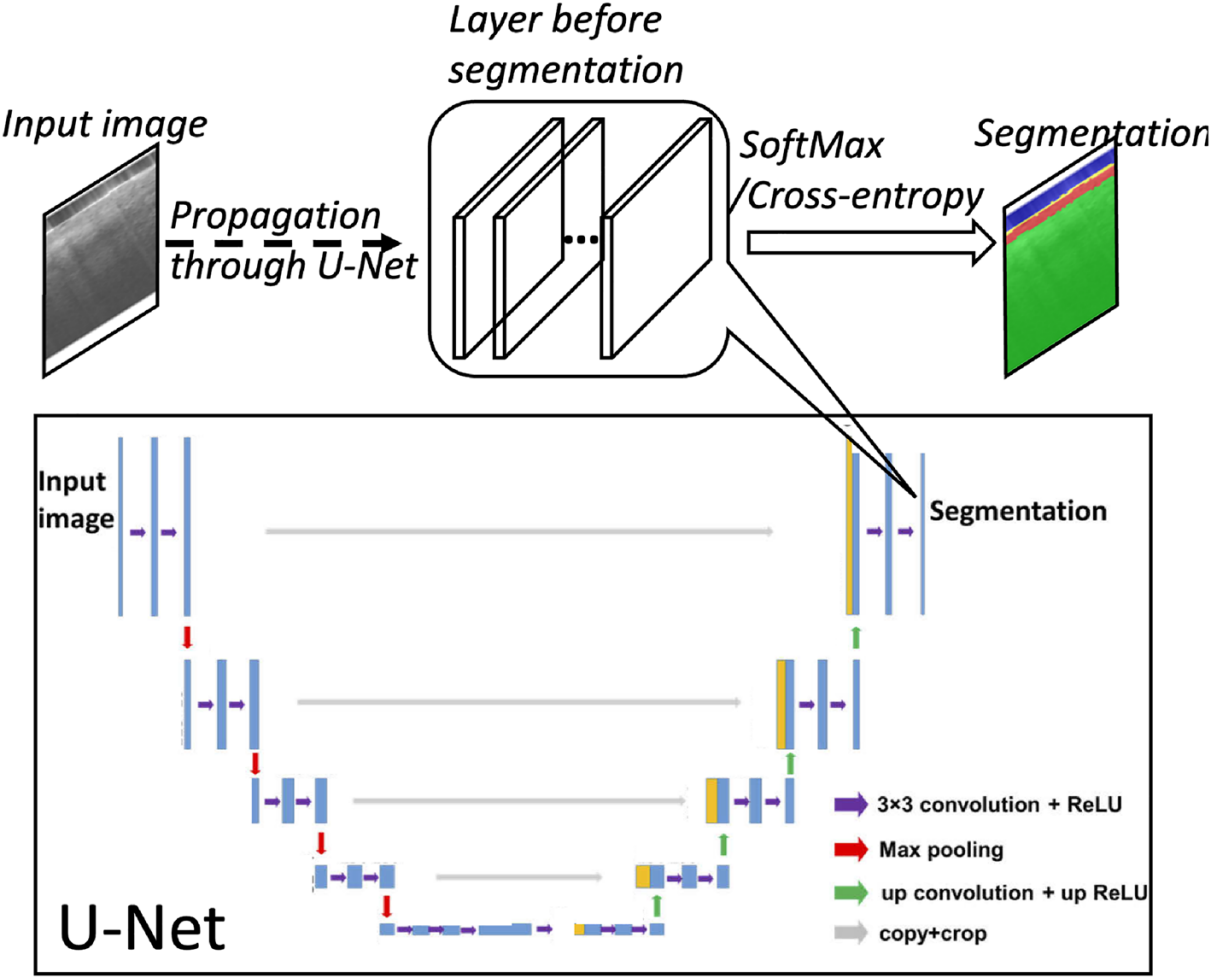
U-Net (bottom), and the use of U-Net activations as features for tissue classification.

**Figure 4:**
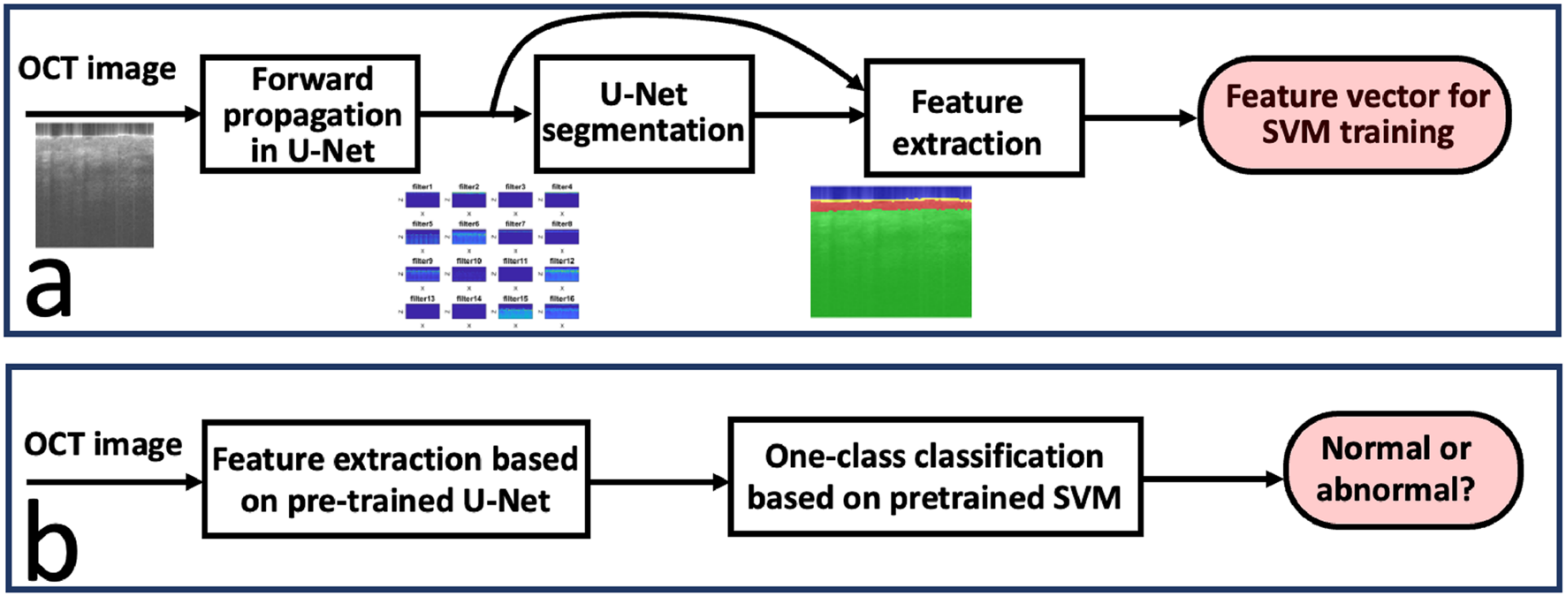
(**a**) Feature extraction using a pre-trained U-Net as a feature extractor; (**b**) classification of an input OCT image using one-class SVM.

We train a one-class SVM classifier to learn the characteristics of normal skin OCT images. The trained classifier is used to detect skin anomaly. It has been demonstrated that one-class SVM classifier separates data in the transformed high-dimensional predictor space to detect outliers and novelties, and is effective at producing decision surfaces from high-dimensional feature vectors^17^. The one-class classification strategy allows us to eliminate the need to acquire images from patients with different skin cancers. The premise of one-class SVM is based on the following facts. First, OCT images of normal skin are similar. Second, OCT images of abnormal skin are different from those of normal skin. To extract features for classification, we forward propagate an input image (*S*_i,j_ where i and j represent pixel index) through the pre-trained U-Net (bottom, Fig. 3) to the layer prior for segmentation (the last ReLu layer of the last decoder stage). At this layer, the network has 16 neurons (N = 16) for each pixel of the image. In other words, the U-Net creates 16 feature for each pixel (top, Fig. 3): *x*_i,j,k_ where k = 1, 2, …, N. To establish feature vectors for each image patch that contains 32 Ascans, we segment the image using the U-Net and average the activations within specific pixel groups: ***X*** = [*X*_1_, *X*_2_, …, *X*_N_] where *X*_k_ = ∑_i_∑_j_ *x*_i,j,k_. The process of feature extraction is summarized in Fig. 4a. By repeating the procedure, we extract feature vectors from different image patches in the training data set. Notably, all the images used for training correspond to normal skin. To determine the skin status, an input OCT image is forward propagated through the U-Net and segmented to create a feature vector. The SVM classifier maps a feature vector to a prediction score and determines whether the skin is normal or abnormal according to the value of the prediction score (positive score: normal tissue; negative score: abnormal tissue), as illustrated in Fig. 4b. Notably, SVM classification is a standard, well established approach. We used SVM tools available in Matlab to develop the classification model. Specifically, the SVM model used in this study is based on a radial basis function (rbf) kernel function with an automatic kernel scale, trained with OCT images of normal skin and annotated with a “normal” label to achieve a specific outlier fraction in one-class classification.

## Data acquisition

### Data acquisition from healthy subjects for U-Net training, feature extraction and classifier training

To train the U-Net and the one-class SVM classifier, we used the OCT imaging platform described in section "OCT imaging system" to acquire images from 9 healthy subjects. We performed in vivo imaging on our subjects, manually scanning a hand-held OCT probe on the skin to obtain 2D images. We did not obtain tissue specimens for ex vivo imaging. Subjects were recruited under an approved IRB protocol (Advarra Pro00035376). All methods involving human subjects were carried out in accordance with relevant guidelines and regulations. All experimental protocols were approved by the Advarra Institutional Review Board. Informed consent was obtained from all subjects. From each subject, we scanned the right forearm, left forearm, forehead, neck, and palm. The age of the subjects ranged from 24 to 59. The skin type of the subjects ranged from type II to type IV. We had both male and female subjects. We excluded images that had low quality and established a training data set. Each image had a dimension of 2048 (Ascan number) by 256 (pixel number in each Ascan). To train the U-Net, pixels of the images were manually labeled to be air (signal free region), stratum corneum, epidermis, and dermis. We divided the images along with the ground truth (results of manual labeling) into smaller patches (32 Ascan per patch), resulting in 2232 image patches. We follow the procedure described in our previous publication to train the network^18^. The U-Net was trained in Matlab 2019b, on graphic processing units (GPU-GTX1070). The training was accomplished in approximately 10 min with an adaptive moment estimation (ADAM) solver, a mini batch size of 40, initial learning rate of 10^−3^, iterated for 10 epochs.

### Pilot imaging study on a patient

To evaluation the trained U-Net and the one-class SVM classifier, we performed a pilot imaging study on a subject with biopsy-confirmed BCC (nodular type). The patient was a 72-year-old male, and the tumor was located on his left jaw. The surgeon first labeled the tumor with a marker, then we performed three sets of scans. First, we scanned the normal skin of forearm of the patient. Then, we scanned the tumor. Lastly, we scanned the region immediately adjacent to the circle draw by the surgeon. The significance of imaging experiment on a patient was to validate if manually scanned OCT imaging coupled with one-class classification allows accurate characterization of skin tissue (normal skin versus skin tumor) on a clinically relevant case. We correlated our classification results with histology to determine if the classification is correct.

## Results

### Selection of features for classification.

As described in section "SVM for one-class classification", we used the U-Net as a feature extractor. Here we assessed different feature selection strategies. To establish a feature vector, we forward propagated the input image through the U-Net up to the layer prior to the segmentation layer (Fig. 3). The chosen layer had N neurons (N = 16) and provided N activation values. For each OCT image patch that had 32 Ascans, we averaged the activations for all the pixels without discriminating pixel type to establish a feature vector, ***X***_all_. Furthermore, we obtained ***X***_e_ by averaging activation values for epidermis pixels determined by the U-Net and obtained ***X***_d_ by averaging activation values for dermis pixels determined by the U-Net. In addition, we established ***X***_e&d_ by concatenating ***X***_e_ and ***X***_d_: ***X***_e&d_ = [***X***_e_;***X***_d_]. When calculating feature vectors, we eliminated pixels categorized as “air” by the U-Net, because these pixels were overwhelmed by noise. Stratum corneum pixels were not considered, because very few pixels were classified as stratum corneum. We extracted feature vectors with respect to different groups of pixels, because pixels belonging to different category (epidermis and dermis) have different characteristics. To evaluate the feature selection strategy, we used 50% of the images in our data set (1116 image patches) to create feature vectors ***X***_all_, ***X***_e_, ***X***_d_, and ***X***_e&d_ for SVM training. For each set of training vectors, we trained a one-class SVM classifier (SVM_all_, SVM_e_, SVM_d_, and SVM_e&d_) using a Gaussian kernel function and a specific outlier ratio.

**Figure 5:**
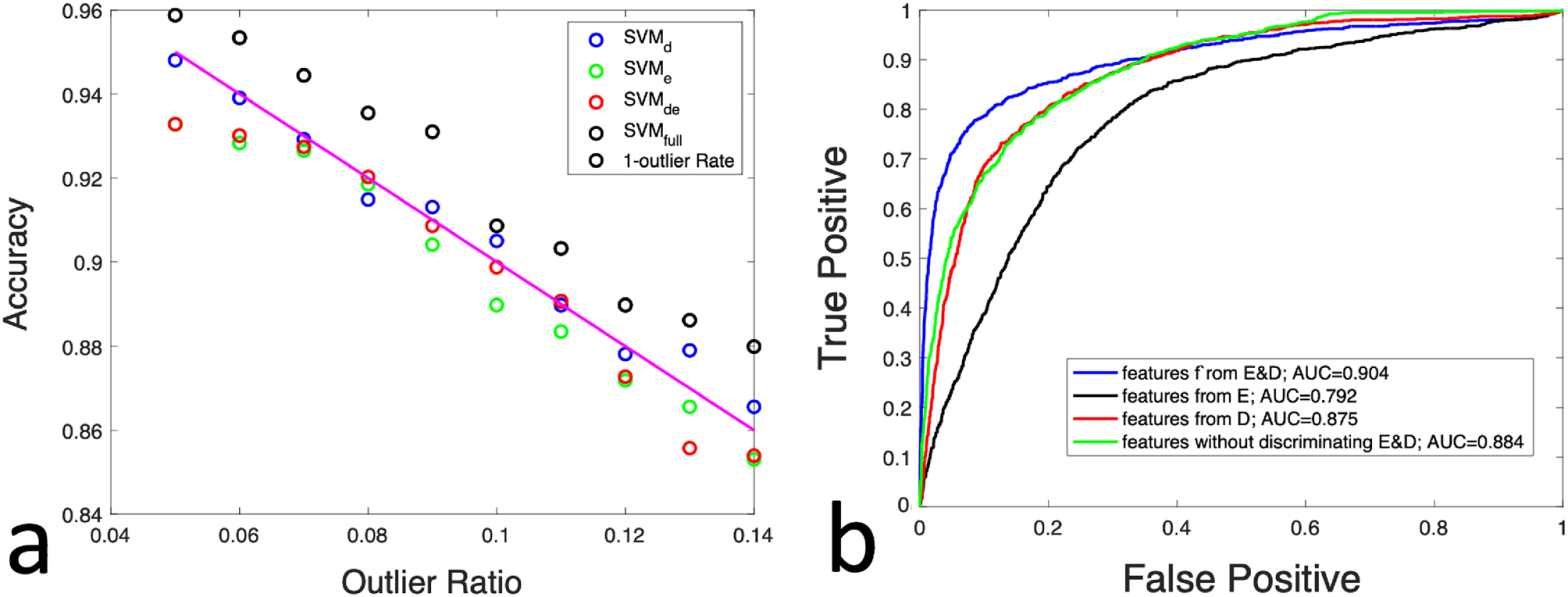
(**a**) Classification accuracy validated using data obtained from normal skin, when the classifiers were trained under different outlier ratios; (**b**) ROC curves for different classifiers, when validated using a data set consisted of normal skin images and computer synthesized abnormal images.

We validated the classification accuracy using the remaining image patches in our data set. We extracted feature vectors from these images, fed the feature vectors to the trained classifiers, determined tissue type according to the output of the classifier, and determined the classification accuracy. Figure 5a shows the one-class classification accuracy obtained by comparing SVM classification with the ground truth (100% normal examples). Figure 5a shows that the classifier trained with a higher outlier ratio tended to classify a larger percentage of testing examples as abnormal.

Furthermore, we created a data set using the image patches (1116 patches) not used for SVM training. The data set had 4464 image patches, including OCT data of normal skin, synthesized BCC data, synthesized SCC image data, and synthesized data with DEJ disruption. We simulated abnormal images representing BCC by reducing OCT signal magnitude to 75% of its original value starting from a random depth within the dermis^21^. We simulated abnormal images of SCC featuring discrete bright regions below the surface, by enhancing the signal magnitude by 25% within a randomly selected region below skin surface^22^. We also created images with disrupted dermis-epidermis junction (DEJ), by normalizing individual Ascans within an image using an averaged depth profile. For each image patch, we created feature vectors (***X***_all_, ***X***_e_, ***X***_d_, and ***X***_e&d_). We labeled a feature vector as “normal” if it was obtained from the normal OCT data, or “abnormal” if it was obtained from synthesized abnormal data (BCC, SCC and DEJ disruption). We fed these feature vectors to the classifiers (trained with an outlier ratio of 8%). We obtained the operating characteristic (ROC) curve shown Fig. 5b and listed the area under curve values for different classifiers in Table 1. We also compared the predictions provided by the classifiers with the ground truth and summarized the prediction accuracies in Table 1. Results in Table 1 suggest that the feature vector that concatenated features of epidermis tissue and dermis tissue (***X***_e&d_ = [***X***_e_;***X***_d_]) outperformed other feature vectors. Therefore, we chose to use ***X***_e&d_ for subsequent classification of experimental data. Using a MacBook Pro computer (Apple M1 CPU and 8 GB RAM) and Matlab R2022a, it takes approximately 0.1 s to extract a feature vector from an image patch with 32 Ascans following the procedure shown in Fig. 4b. It takes approximately 0.01 s for the SVM classifier to make the prediction.

**Table 1:** Assessment of SVM classification when the classifiers were trained with an outlier ratio of 8%.

	SVM_e&d_	SVM_e_	SVM_d_	SVM_all_
AUC of ROC	0.91	0.79	0.88	0.88
Accuracy	0.69	0.55	0.68	0.65

### Spatially resolved tissue classification based on one-class tissue classification

**Figure 6:**
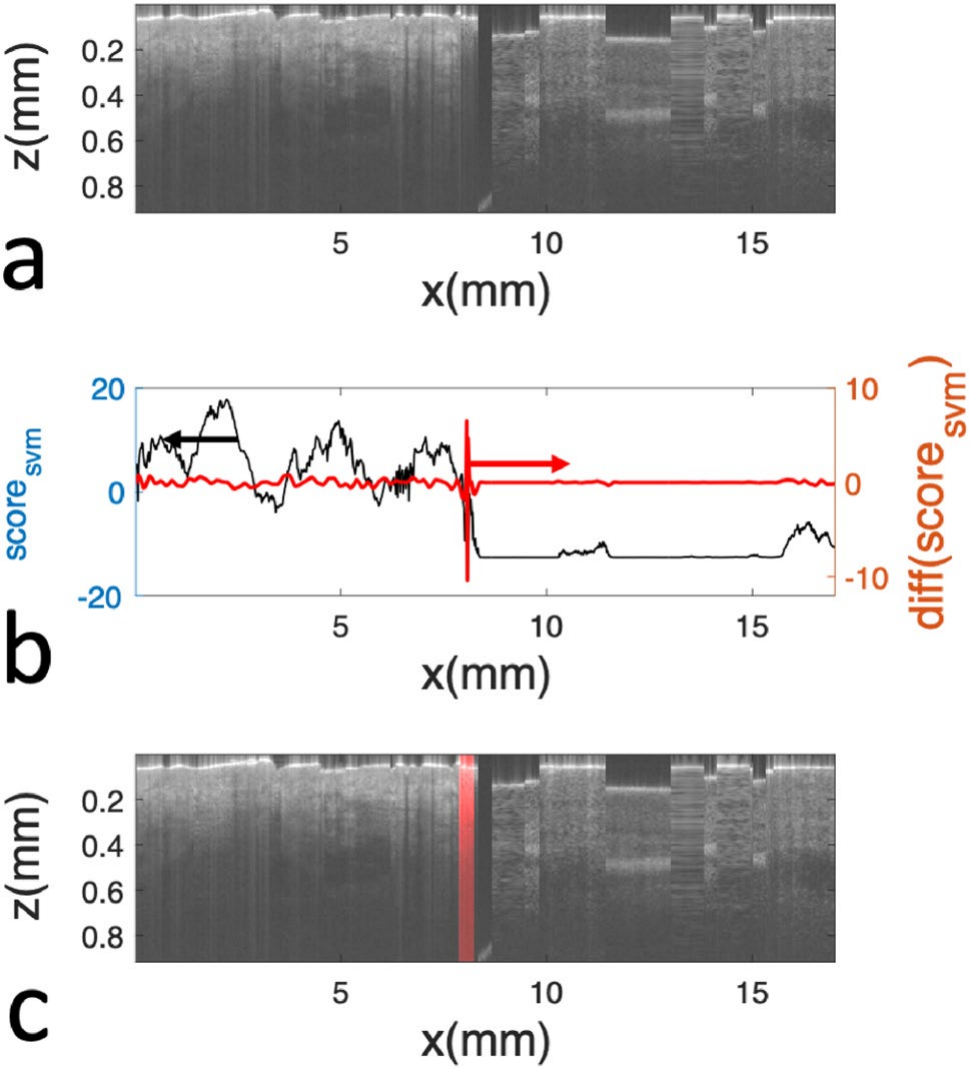
(**a**) OCT image obtained by scanning the fiber-optic probe across the junction between the skin and the nail plate from a healthy subject; (**b**) spatially resolved SVM prediction score (black curve), and first order difference of filtered prediction score (red curve); (**c**) boundary between skin and anormal tissue (red shade) identified through one-class SVM classification.

To demonstrate how the one-class classifier allowed spatially resolved tissue classification, we scanned the fiber-optic OCT probe from the skin to the nail plate at the thumb of a healthy subject. The image obtained is shown in Fig. 6a. The left side of the image corresponds to the skin and the right side of the image corresponds to the nail plate. The OCT signal obtained from the nail was different from that of the skin and was considered as abnormal. For an image patch at a specific lateral coordinate, we extracted features from epidermis pixels and dermis pixels, and concatenated these features to establish ***X***_e&d_. Using the pre-trained one-class SVM classifier SVM_e&d_, we were able to obtain prediction scores at different spatial locations (Fig. 6b, black curve). To determine the edge between normal skin and anomaly (nail plate), we filtered the SVM prediction scores (thresholding in wavelet domain) and obtained the first order difference of the filtered SVM prediction score (red curve in Fig. 6b). The peak location of the red curve corresponds to the boundary between normal and abnormal tissue, where the SVM prediction score changes abruptly. The boundary location is shaded with the color of red in Fig. 6c, suggesting that one-class SVM using features extracted from both epidermis and dermis allowed spatial resolved tissue classification and tissue boundary detection.

### Pilot patient study.

**Figure. 7:**
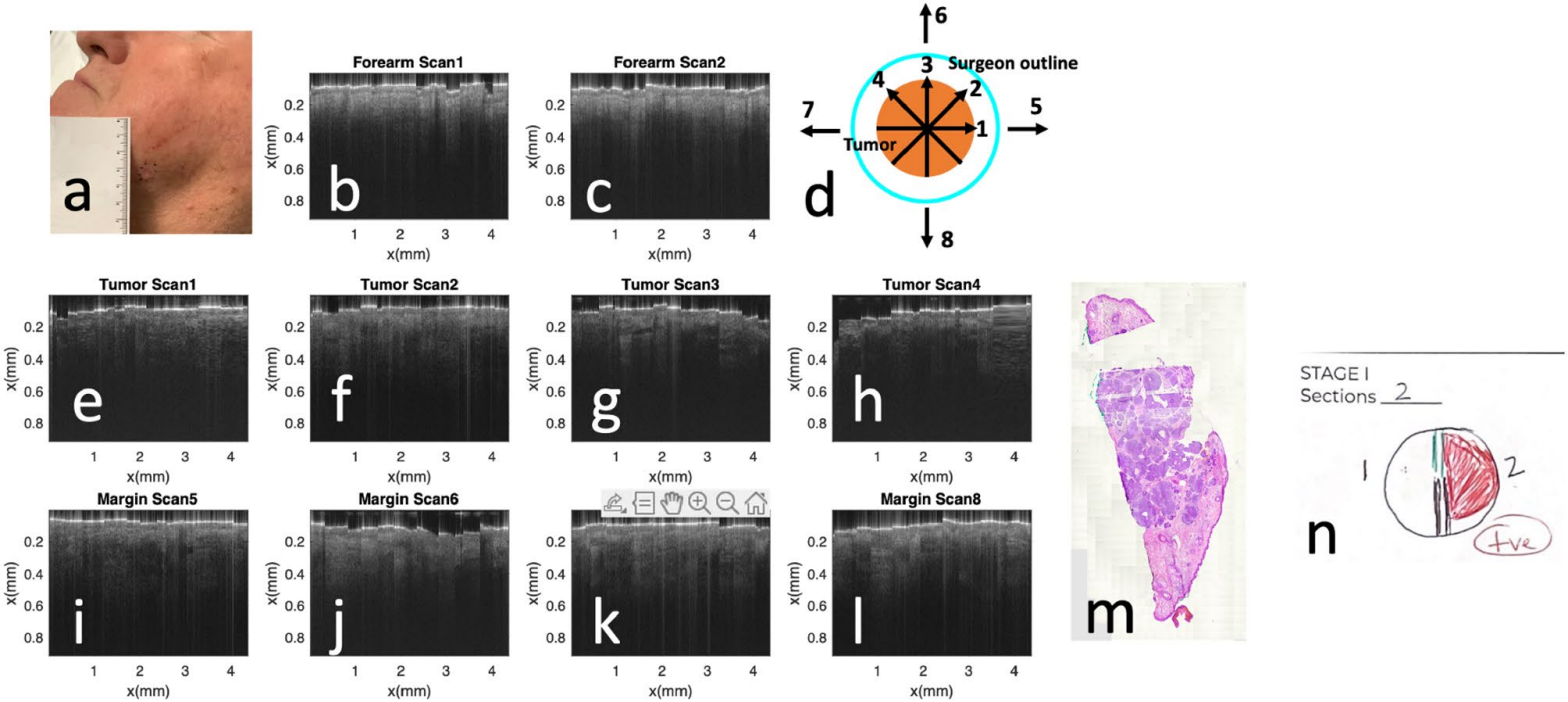
(**a**) Clinical photo taken from the patient; (**b,c**) OCT image obtained from the forearm of the patient; (**d**) scanning pattern used to profile the tumor; (**e–h**) OCT images obtained following trajectories 1–4 in Fig. 6d; (**i–l**) OCT images obtained following trajectories 5–8 in Fig. 6d; (**m**) result of histology examination; (**n**) Mohs histology documentation, depicting histologically positive Stage 1 margins.

In a pilot clinical imaging experiment, we imaged a 72-year-old male patient with biopsy-confirmed BCC (nodular type) located on his left jaw (Fig. 7a). To benchmark OCT images of the tumor against normal skin image, we obtained OCT images of normal skin from two different locations at the forearm of the patient (Fig. 7b,c). In OCT images obtained from the normal skin of the patient, the first layer of the skin (stratum corneum) is thin and bright, followed by the epidermis with reduced brightness and clearly visible DEJ. Underneath is the dermis where the signal decreases as depth. We scanned the tumor following trajectories 1–4 shown in Fig. 7d, and show the images obtained in Fig. 7e–h. Compared to the normal skin from the same patient, images obtained from the tumor show disruption of DEJ and reduced OCT signal amplitude starting from upper dermis. We also scanned the region immediately adjacent to the circle drawn by the surgeon, following trajectories 5–8 shown in Fig. 7d. Images obtained are shown as Fig. 7i–l. Notably, every OCT image shown in Fig. 7 has 256 Ascans, corresponding to a ~ 4.4 mm lateral scanning range. A smaller lateral range was chosen to ensure that Fig. 7e–h were obtained from the tumor without ambiguity. To perform one-class tissue classification, we divided an OCT image into eight non-overlapping patches (32 Ascans per patch). For every image patch, we followed the procedures illustrated in Fig. 4a to establish feature vectors for different image patches, and used the pre-trained one-class SVM classifier to output a prediction score. A positive prediction score corresponded to normal skin tissue, while a negative prediction score corresponded to abnormal skin tissue. We averaged the score using results from all the 8 patches within an image, and summarized the results in Table 2. For OCT images obtained from the forearm skin (normal) and OCT images obtained from the tumor (abnormal), the one-class classifier predicted the tissue to be normal and abnormal, respectively. The tissue classification was correct. On the other hand, scans performed outside of the circle drawn by the surgery (Scan 5–8 in Fig. 7d, images in Fig. 7i–l) provide margin assessment. According to the results of one-class SVM classification, Fig. 7i,j (Scan 5 and Scan 6 in Fig. 7d) correspond to abnormal skin, in other words, the margin was positive. Figure 7k,l (Scan 7 and Scan 8 in Fig. 7d) correspond to normal skin. In other words, the margin was negative. To validate the margin assessment results, we show the result of histology examination in Fig. 7m,n. Histology suggested a positive margin, which is consistent with our classification results,

**Table 2:** One-class SVM classification of OCT images obtained from a BCC patient.

	SVM score	Normal or abnormal
Normal skin (forearm site 1)	4.2	Normal
Normal skin (forearm site 2)	2.1	Normal
Tumor (scan 1)	–5.3	Abnormal
Tumor (scan 2)	–3.2	Abnormal
Tumor (scan 3)	–5.3	Abnormal
Tumor (scan 4)	–5.3	Abnormal
Margin (scan 5)	–2.3	Abnormal (positive margin)
Margin (scan 6)	–4.8	Abnormal (positive margin)
Margin (scan 7)	2.9	Normal (negative margin)
Margin (scan 8)	6.5	Normal (negative margin)

## Conclusion and discussion

The overall aim of this study is to perform in vivo skin characterization through manually scanned OCT imaging and machine learning algorithms for automatic tissue classification. The role of the proposed algorithm is to determine whether the skin under OCT imaging is normal or abnormal. In this study, we investigated a method for automatic skin tissue characterization based on OCT imaging. The focus of this study was to develop a novel machine learning classification method to automatically identify abnormal skin tissues through one-class classification. Our approach addressed two challenges in automatic tissue classification. First, feature engineering is challenging for OCT data that is complicated and affected by speckle noise. Second, OCT data obtained from skin tumor show a wide range of characteristics, depending on tumor type, stage, and other factors (patient skin type and age). To train a machine learning algorithm for automatic skin cancer detection, it requires a massive training data set that has sufficient examples to represent the diversity of features for different cancers. To effectively determine the boundary of tumor during Mohs surgery, our method does not intend to determine the type of the tumor which is confirmed through biopsy prior to the surgery. Instead, we used a U-Net as a feature extractor and trained one-class SVM classifiers using data of normal skin tissue. The algorithm learns the features of normal skin tissue and reports anomaly when it encounters features different from normal features. We validated the effectiveness of the one-class classification method, using computer synthesized data, and experimental data. Our results showed that the one-class classifier allowed accurate detection of abnormal skin tissue for synthesized OCT data of BCC, SCC and disrupted DEJ, and OCT data from nail plate. We demonstrated spatially resolved tissue classification (Fig. 6) when the classifier was applied to OCT image patches obtained at different locations. Results of one-class classification correlated well with histology (Table 2 and Fig. 7) spatially, thanks to the effectiveness of the algorithm and the small dimension of the instrument. The one-class classifier is able to identify normal and abnormal skin at different body regions, as long as the training data set has examples from different body regions. In this study, we trained the one-class classifier using images from normal skin at different locations (forearm, left forearm, forehead, neck, and palm). We can acquire data for algorithm training from other body locations to improve the robustness of the classifier.

Our approach is the combination of a deep learning model (U-Net) and a traditional machine learning model (SVM). First, compared to traditional machine learning classifiers that use manually established features, our method is unique as it uses features automatically extracted from the deep learning model. Second, typical deep learning neural network models consider two or more classes, optimizes a cross-entropy (or other measures) to determine weights and biases for the model, and are usually not compatible with one-class classification. In comparison, our approach performs one-class SVM classification, training the deep learning network and the classifier using data of normal skin. Our method effectively overcome challenges such as limited numbers of images obtained from skin tumors and diverse features associated with abnormal skin.

The algorithm extracts a feature vector for an image patch. and classifies the image patch to be normal or abnormal. The spatial resolution of our classification method depends on the size of image patch, because the feature vector is obtained by averaging activation values of pixels within the image patch. In our current method, each image patch used in classification has 32 Ascans (~ 0.54 mm). When smaller patches are used to train the classifier and predict tissue type, the localization precision may be improved, probably at a cost of classification accuracy.

The U-Net used as feature extract has 16 features in the layer before segmentation. We extract features from epidermis pixels and dermis pixels respectively and establish a concatenated feature vector with 32 features for one-class classification. Classification based on more features may achieve a higher accuracy, but is likely to be affected by overfitting and has limited robustness.

OCT image data is inevitably affected by noise. A noise reduction pre-processing step might enhance the performance of the classifier under certain conditions. However, noise reduction may also compromise the robustness of the algorithm. First, OCT images are largely affected by speckle that appears as random modulation and is considered as the major noise source for OCT. However, speckle carries sub-resolution features of the sample and has been used as a feature for tissue classification. A generic noise reduction method is likely to suppress speckle which is in fact a discriminating feature. Second, the features extracted from the U-Net are at different scales. A non-local feature is calculated using OCT signals at different spatial locations and is inherently noise suppressing. Third, when a feature is significantly affected by noise, its contribution to the SVM classifier is minimized following the training process. Hence, we chose to use OCT data without noise reduction to train a robust classifier.

## Data Availability

Data underlying the results presented in this paper are not publicly available at this time but may be obtained from the corresponding author upon reasonable request.
